# Tension Pneumocephalus Induced by Nasal Cannula

**DOI:** 10.1155/2019/2757561

**Published:** 2019-02-17

**Authors:** Hao Xiao, Tony Chen, Andrew Tagg

**Affiliations:** ^1^Department of Emergency Medicine, Western Health, Footscray, Victoria, Australia; ^2^Department of Radiology, Western Health, Footscray, Victoria, Australia

## Abstract

Tension pneumocephalus is a rare medical emergency. Spontaneous atraumatic tension pneumocephalus is reported in cases of neoplasm, Valsalva manoeuvres, and air cell hyperpneumatization. For the first time, we describe a case of atraumatic tension pneumocephalus induced by nasal cannula in a patient with ventriculoperitoneal shunt. Our case report discusses the possible mechanisms of the tension pneumocephalus in this case.

## 1. Introduction

Pneumocephalus is defined as intracranial gas collection in the epidural, subdural, subarachnoid, intraventricular, or intraparenchymal spaces. It is commonly caused by facial trauma, ear infections, or neurosurgical interventions [1]. Cases of spontaneous nontraumatic pneumocephalus are highly uncommon with previously reported neoplasm, barotraumas, Valsalva manoeuvres, and air cell hyperpneumatization [2]. To our knowledge, no case has described nasal cannulas as a potential source of spontaneous pneumocephalus. We present here an interesting case of atraumatic tension pneumocephalus in a patient with a ventriculoperitoneal shunt newly started on a nasal cannula

## 2. Case Report

A 74-year-old man from a high-care-level nursing home was brought in by ambulance with a sudden decrease of GCS that had persisted for one day. Collateral history from the nursing home revealed that the patient had developed upper respiratory tract symptoms including cough and a runny nose in the past week. He was reviewed by a local general practitioner 4 days prior because of desaturating on room air. The local general practitioner started the patient on a 3 L nasal cannula and Augmentin Duo Forte. The patient initially recovered well with nasal oxygen and was active in the nursing home. However, a dramatic decline in his consciousness in the morning left him unarousable, which pressed nursing home staff to seek urgent medical help.

**Figure 1:**
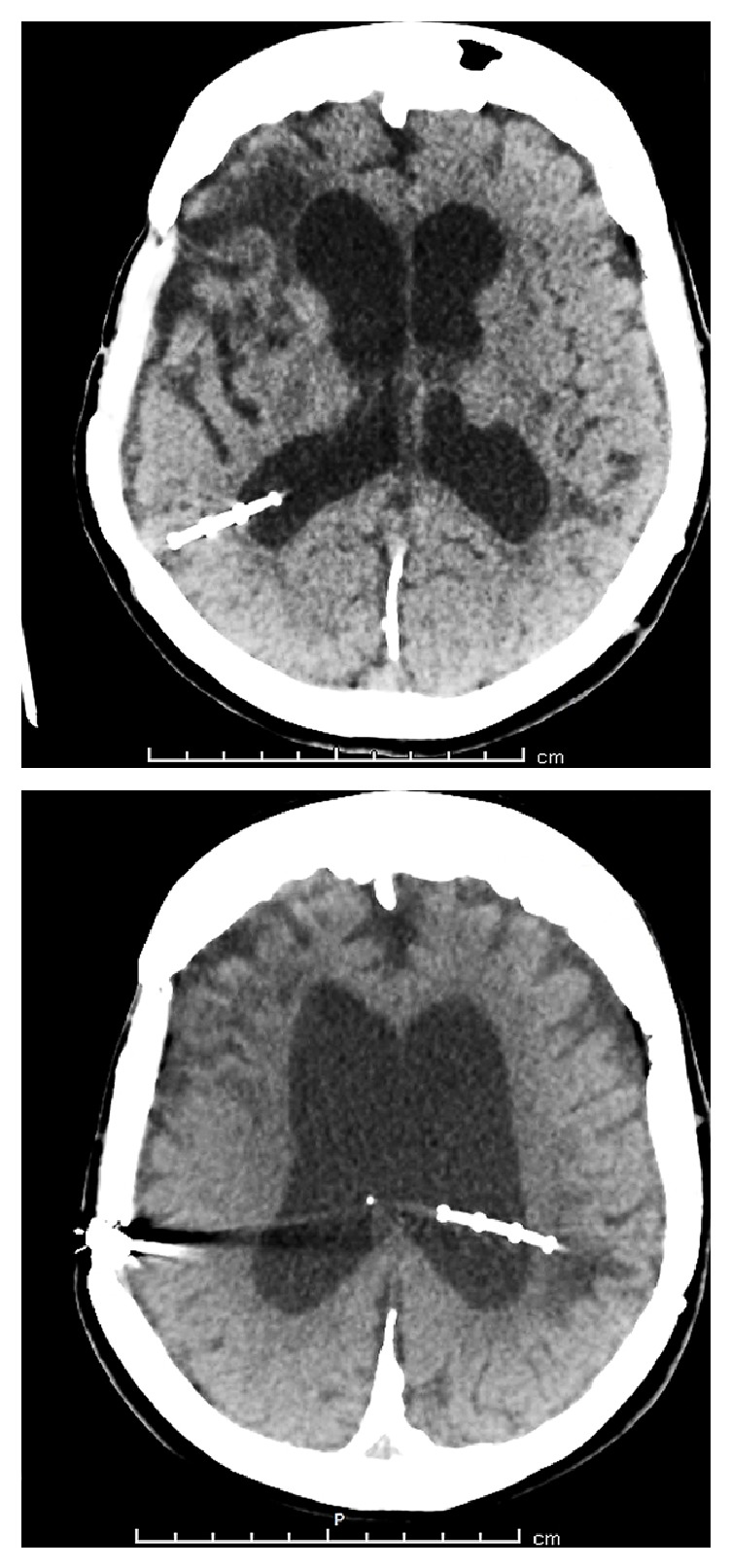
CT-brain scan demonstrates no intracranial free air and VP shunt in place in patient's 2 months prior presentation.

The patient had a background of acquired brain injury and normal pressure hydrocephalus; a ventriculoperitoneal (VP) shunt was inserted 30 years ago. Other past histories included epilepsy, hypertension, advanced dementia, and schizoaffective disorder. He was admitted into the same hospital 2 months prior because of delirium secondary to community-acquired pneumonia. At the time, a CT-brain scan showed bilateral VP shunts in place and no acute intracranial pathology (Figure 1). The patient is usually verbal and mobile with a 4-wheel frame walker at the nursing home.

**Figure 2:**
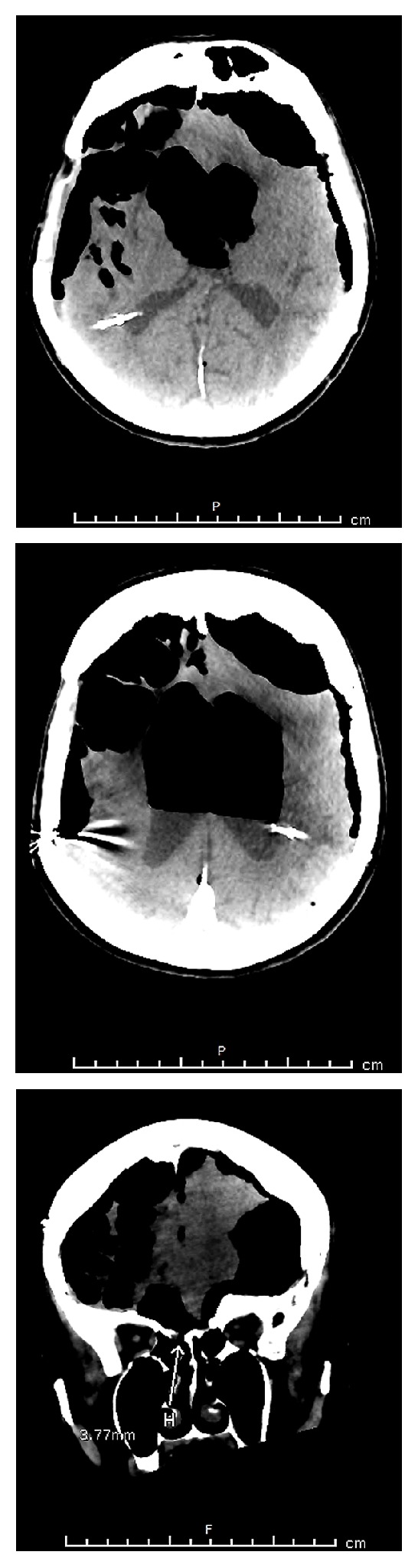
CT-brain scan shows large amount of intracranial free air and bony defect at the right cribriform plate after starting the patient on nasal cannula.

On admission, the patient's GCS was recorded as 9/15 E4, V1, M4. A CT scan demonstrated a massive volume of intracranial gas with positive pressure effect within the lateral and third ventricles. The CT scan of the base of the skull also revealed a small bony defect at the right cribriform plate with gas traversing from the nasal cavity to the cranium (Figure 2).

Unfortunately, due to his comorbidity and high anaesthetic risks, the neurosurgical team deemed the patient unsuitable for operation. He was conservatively managed with high-flow nasal oxygen and subsequently transferred to and palliated in a nursing home.

## 3. Discussion

A previous review of 295 pneumocephalus cases by Markham in 1967 indicated that trauma is the most common etiological factor of pneumocephalus, accounting for 74% of all cases [3]. Neoplasm and infection are the second and third highest causes, found in 13% and 9% of cases, respectively [3]. Of the remaining 4% (13 cases), 11 were reported to be due to surgical complications, and 2 cases did not have any known etiology [3]. Other authors have commonly reported barotrauma as the cause of spontaneous pneumocephalus [4, 5]. Noninvasive ventilation creates high pressure beyond normal intracranial pressure and can drive air into the intracranial cavity [4, 5]. Our case report provided a rare case of normal pressure device (i.e., nasal cannula) induced tension pneumocephalus.

Two theories have been proposed to explain the pathophysiology of pneumocephalus. One theory is the “ball valve mechanism.” Fragments of bone and dural flap can act as unidirectional valves that trap air within the intracranial cavity under higher external pressure [5]. Another mechanism suggested explaining pneumocephalus as the “inverted bottle effect mechanism.” This mechanism is usually associated with cerebrospinal fluid leakage, which creates negative intracranial pressure; compensatory air enters the cranium to equalize the pressure [5]. In our case, the spontaneous pneumocephalus was the result of both mechanisms. The existing ventriculoperitoneal shunt created passage for CSF to enter peritoneal space and predisposed the patient to pneumocephalus. With the additional increase of external pressure, such as a nasal cannula, air enters into intracranial space. The cribriform plate defect was the passage for the air entry and also served as the “ball valve,” trapping air within the intracranial space.

Treatment for pneumocephalus can be conservative therapy or surgical intervention depending on the underlying cause of air entrapment and severity. Tension pneumocephalus producing significant symptoms is considered a neurosurgical emergency and must be evacuated promptly [6]. The treatment options include placement of a burr hole, needle aspiration, and closure of dura defect [6]. In our case, the patient was not a surgical candidate; therefore, he was managed conservatively. Conservative treatment involves placing the patient in the Fowler position of 30°, avoiding the Valsalva manoeuvre, and administering osmotic diuretics [7]. Another novel conservative therapy for pneumocephalus consists of augmenting patient oxygenation (i.e., using a high-pressure nasal cannula). Seigel et al. reported 3 cases in which patients with symptomatic pneumocephalus benefited from high-flow nasal cannulas [8]. The theory is that augmented oxygenation can wash out pulmonary nitrogen, creating a gradient in which nitrogen in the intracranial air bubble can diffuse into the blood [8]

## 4. Conclusion

Pneumocephalus of nontraumatic, spontaneous origin is rare. Our case suggests that a nasal cannula can induce tension pneumocephalus in a patient with a ventriculoperitoneal shunt. Using nasal cannulas should be carefully considered when a patient has had previous surgical procedures that reduce intracranial pressure.
